# Investigation of cytotoxic effect and action mechanism of a synthetic peptide derivative of rabbit cathelicidin against MDA-MB-231 breast cancer cell line

**DOI:** 10.1038/s41598-024-64400-1

**Published:** 2024-06-12

**Authors:** Marzieh Bashi, Hamid Madanchi, Bahman Yousefi

**Affiliations:** 1 Department of Immunology, Semnan University of Medical Sciences, Semnan, Iran; 2 Cancer Research Center, Semnan University of Medical Sciences, Semnan, Iran; 3 Nervous System Stem Cells Research Center, Semnan University of Medical Sciences, Semnan, Iran; 4 Department of Biotechnology, School of Medicine, Semnan University of Medical Sciences, Semnan, 35131-38111 Iran; 5 Drug Design and Bioinformatics Unit, Medical Biotechnology Department, Biotechnology Research Center, Pasteur Institute of Iran, Tehran, 13198 Iran

**Keywords:** Cathelicidin, Antimicrobial peptides, Anticancer peptides, Cap18, Biochemistry, Biological techniques, Biotechnology, Cancer, Cell biology, Molecular biology

## Abstract

Antimicrobial peptides (AMPs) have sparked significant interest as potential anti-cancer agents, thereby becoming a focal point in pursuing novel cancer-fighting strategies. These peptides possess distinctive properties, underscoring the importance of developing more potent and selectively targeted versions with diverse mechanisms of action against human cancer cells. Such advancements would offer notable advantages compared to existing cancer therapies. This research aimed to examine the toxicity and selectivity of the nrCap18 peptide in both cancer and normal cell lines. Furthermore, the rate of cellular death was assessed using apoptosis and acridine orange/ethidium bromide (AO/EB) double staining at three distinct incubation times. Additionally, the impact of this peptide on the cancer cell cycle and migration was evaluated, and ultimately, the expression of cyclin-dependent kinase 4/6 (CDK4/6) genes was investigated. The results obtained from the study demonstrated significant toxicity and selectivity in cancer cells compared to normal cells. Moreover, a strong progressive increase in cell death was observed over time. Furthermore, the peptide exhibited the ability to halt the progression of cancer cells in the G1 phase of the cell cycle and impede their migration by suppressing the expression of CDK4/6 genes.

## Introduction

Cancer treatment, especially breast cancer, has undoubtedly become a serious global public health challenge to the rising incidence and mortality rate^1^. Current approaches, such as surgery, radiotherapy, chemotherapy, or a combination, can help prolong life expectancy^2^. Although, several barriers would affect and restrict their efficiency^3^. Besides, several anti-cancer drugs have serious adverse effects on normal cells due to their lack of selectivity^4,5^.

Working on a specialized approach to tumors, cancer treatment nowadays shifted to molecular targeting to develop alternative cancer therapy^6^. To find novel anti-cancer approaches and therapies, antimicrobial peptides (AMPs) have attracted much attention. As required components in almost all microorganisms' innate immune systems, it has been anticipated to surpass traditional medicine's limitations even more^7^. While most clinical research focused on the potential usage of antimicrobial effects of AMPs, many other recent studies revealed their anti-cancer functions^8^; hence, these peptides are termed anti-cancer peptides (ACPs)^9^. In recent years, research on potential treatments for various cancerous cell lines, such as breast, lung, melanoma, and lymphoma, has elucidated the cytotoxic effects of various ACPs from different sources^10^. From another point of view, ACPs have unique characteristics, such as short half-life in vivo, ease of synthesis and modification, low toxicity against normal cells, and biocompatibility^4,10–12^.

Therefore, it would be worthwhile to design more potent and more selective ACPs that exhibit a variety of mechanisms of action against human cancer cells and offer significant advantages over current therapies^8^. Despite the possibility that AMPs will find clinical utility, several obstacles stand in the way of their clinical application, such as collateral toxicity on normal mammalian cells and severe immunological reactions^13^. Another limitation that needs to be overcome is their synthesis cost. To generate economic ACPs, redesigning and modifying them using In-silico methods would be helpful to preserve their advantages and simultaneously reduce their collateral toxicity^14^.

However, considering the two opposite faces of cathelicidin in cancer development, modifying AMPs to ACPs has become one of the innovation strategies in favor of combating cancer cells^15^. With the help of designing and modifying native peptides (especially, cathelicidin) via in silico method, it is now possible to amplify desired properties and synthesize stronger anticancer peptides^16^. Many previous studies^6,17–21^ on cancer cell lines related to modified derivative cathelicidin peptides, particularly human cathelicidin, indicated several potential anticancer properties of these peptides; some were also studied on animal models^22^. Hence, it could be considered that modified rabbit cathelicidin peptides could have various potential properties like anticancer effects.

The nrCap18, currently evaluated and discussed, is a new modified cationic peptide derivative based on rabbit CAP18 with good lipopolysaccharide (LPS) binding activity and proper antimicrobial properties. According to a previous study^23^, nrCap18 targets the cell membranes and walls with a negative charge such as bacteria. So, it would be concluded that cancerous cells with negative charge membranes seem to be a good target to be selectivity attacked. Here, we investigated some anticancer properties of nrCap18 on the MDA-MB 231 cell line, a triple-negative breast cancer (TNBC) cell, for the first time and provided insight into the mechanism and cytotoxicity of this peptide.

## Material and methods

### Chemicals and reagents

DMEM (Dulbecco's Modified Eagle Medium) High Glucose, FBS (Fetal bovine serum), and EDTA (Ethyl Diamine Tetra Acetic acid) were purchased from Gibco (New York, USA). 2-(4-(2-hydroxyethyl) piperazine-1-yl) ethane sulfonic acid, Penicillin and streptomycin, trypan blue dye, trypsin, MTT dye (3-(4,5-dimethylthiazol-2-yl)-2,5-diphenyltetrazolium bromide), glutaraldehyde, and DMSO (dimethyl sulfoxide) were purchased from Sigma-Aldrich (St. Louis, MO, USA). Total RNA Purification Kit and cDNA synthesis kit were purchased from Jena Bioscience, (Jena, Germany). For Real-time PCR, Real Q Plus 2 × Master Mix Green made by the AMPLIQON (Odense, Denmark) was used.

### Culture conditions and cell lines

The triple-negative breast cancer cell line MDA-MB 231 (ATCC HTB-26) and the normal breast cell line MCF-10A (ATCC CRL-10317™), were initially obtained from the National Cell Bank of Iran (Pasteur Institute, Iran). The MDA-MB 231 cell line grew in DMEM-High Glu (medium supplemented with 15 mM of HEPES (2-[4-(2-hydroxyethyl) piperazin-1-yl] ethane sulfonic acid), 24 mM sodium bicarbonate, 1% penicillin–streptomycin, pH 7.4, and 10% fetal bovine serum.

MEBM (Mammary Epithelial cell Basal medium), which is included in the MEGM (Mammary Epithelial cell Growth medium) Bullet Kit (available from Clonetics Corporation, Cat. No. CC-3150) is the base medium used for the MCF-10A cell line. To make the complete growth medium, the following components were added to the base medium: All MEGM SingleQuot additives that are supplied with the kit )the GA-1000 (BPE (Bovine Pituitary Extract) 13 mg/ml, 2ml; hydrocortisone 0.5 mg/ml, 0.5 ml; hEGF (human Epithelial Growth Factor) 10ug/ml, 0.5; insulin 5 mg/ml, 0.5 ml)( except Cholera toxin 100 mg/ml (sold separately-Sigma Aldrich).

The culture conditions for the cells were 37 °C, 5% CO_2_, and 90% humidity; also, they were passaged using trypsin/EDTA and PBS (phosphate-buffered saline) solution.

### Peptide synthesis

nrCap18 peptide with WRKRLRKFRLKKKILQ sequence and CLIP peptide (Class II-associated invariant chain peptide) as negative control with PVSKMRMATPLLMQA sequence were produced using a solid-phase synthesis technique under Mimotopes' (Mulgrave, Australia) fluorene-9-methoxycarbonyl (Fmoc)-polypeptide active ester chemistry. The synthesized peptides were purified to 95% using the RP-HPLC (Reverse Phase-High Pressure Liquid Chromatography) method. The Sciex API100 LC/MS (Liquid Chromatography/Mass Spectrometer) mass spectrometer (Massachusetts, USA) was used to confirm the molecular weight and sequence accuracy of the peptides.

### MTT assay

In a 96-well plate, 5 × 10^3^ cell/well MDA-MB 231 and 10^4^ cell/well MCF-10A cells were incubated overnight in different concentrations (1000, 500, 50, 5, and 0.5 μg/ml) nrCap18, various concentrations (100, 10, 1 and 0.1 μg/ml) Doxorubicin and complete medium as a negative control for 24 h. A 5 mg/ml stock solution of “MTT” (1-(4,5-Dimethylthiazol-2-yl)-3,5-diphenyl formazan, Thiazolyl blue formazan) was made in sterile PBS, and 10 μl of the cell culture medium was added to each well that held 100 μl of it. The MTT-containing medium was taken out after 4 h and 100 μl of sterile DMSO was added to solubilize the formazan crystal^23^. The solutions were analyzed in an ELISA plate reader (BIOTEK Inc, USA), measuring the absorbance at 570 nm, then analyzed on GraphPad Prism 9.0 software to determine the 50% inhibition concentration (IC_50_). The following equation was used to determine the percentage of cell viability based on the absorbance readings of the test and control wells^24^.

$$\% Cell\;viability = \left[ {\left( {Mean\;absorbance\;in\;test} \right)/\left( {Mean\;absorbance\;in\;control} \right)} \right] \times 100$$

$$\% \, Toxicity \, = \, 1 - \, Viability \, \%$$

### Evaluating the nrCap18 selectivity using MTT after washing method

The selectivity of the nrCap18 to the cells to the cancer cell membrane (MDA-MB 231 cell line) was evaluated by the MTT assay with some modification called MTT after the washing method, which was previously explained by Madnachi et al.^25^ nrCap18 peptide has a high affinity to the negative charge cell membranes (cancer cells) due to its high cationic property, meanwhile, it has less affinity to normal cells. At first, the MDA-MB 231 and MCF-10A cells were cultured in a 96-well plate overnight. The next day, after depletion of the supernatant, the cells were incubated with various concentrations (1000, 500, 50, 5, and 0.5 μg/ml) of nrCap18 and complete medium as a negative control for an hour. After an hour of treatment, the wells were drained and washed twice with PBS. Next, the complete medium was added to wells, and the incubation continued for 24 h. Finally, other steps were performed as in a standard MTT method^25^.

### Determination of apoptosis by flow cytometry

Cell apoptosis was assayed using the Annexin V-FITC/7-AAD detection kit (BioVision, Inc). MDA-MB 231 cells were seeded in 6-well plates and incubated overnight. After that, the cells were treated with IC_50_ concentrations of nrCap18 and Doxorubicin for 24 and 48 h before being harvested following trypsinization and centrifugation. An automatic hemocytometer (Countess II, ThermoFisher, USA) was used to count the number of cells, and the cell density was adjusted to approximately 1 × 10^6^ cells/ml. Following the manufacturer’s recommendations (BioVision, Inc.), samples were resuspended in 500 μl binding buffer after being twice washed with PBS. After adding 5 μl Annexin V-FITC and 5 μl 7-AAD, the cell mixture was incubated at room temperature for five minutes. A flow cytometer by BD FACSCalibur (BD Biosciences, California, USA) was used to analyze the samples. Annexin V^+^, 7-AAD^−^ cells were considered in the early stage of apoptosis, while Annexin V^+^, 7-AAD^+^ cells were supposed to be in the late stage of apoptosis^25^. At last, data was analyzed by FlowJo software (version 10, TreeStar, USA).

### Live/dead double staining

To evaluate the effects of the nrCap18 peptide on the cell membrane, an acridine orange/Ethidium Bromide (AO/EB) double staining assay was conducted. In brief, the cells were seeded in 6-well plates and incubated overnight for adhering. After being cultured for 72 h with the peptide at the relevant IC_50_ doses, samples were washed with PBS twice. Next, 20 μl of trypsin was added to each well. Following cell sloughing, suspensions (25 μl) were put onto glass slides. After adding 1 μl of a dual fluorescent staining solution with equal concentrations of 100 μg/ml of Ethidium Bromide (EB) and Acridine Orange (AO) to each suspension, a coverslip was placed over the mixture, and the UV-fluorescence microscope was used to image the samples within 30 min of the fluorescence fading off. The morphology was examined using a fluorescent microscope and repeated three times at least^23^.

### Analysis of the cell cycle using propidium iodide (PI)

An assessment of the impact of nrCap18 on the MDA-MB 231 cell cycle has been done using a cell cycle arrest test. IC_50_ dose of nrCap18 was used to inoculate treated cells, while control cells (untreated cells) were cultured in DMSO at 20 mg/ml. Following two PBS washes, harvested tumor cells were resuspended at 3 × 10^6^ cells/ml in a cell suspension buffer (PBS + 2% fetal bovine serum, or FBS; PBS + 0.1% bovine serum albumin, or BSA). The tumor cells were spun at 300 × g for 5 min and the supernatant was discarded. 5 ml of cold 70% ethanol was added dropwise to cell suspensions in 500 μl buffer that had been aliquoted in 15 ml V-bottomed polypropylene tubes using a moderate vortex. Before flow cytometric analysis and propidium iodide (PI) staining, cells were fixed for at least an hour at 4 °C. PBS was used twice to wash the fixed cells. 1 ml of PI staining solution at 50 μg/mL was added to the cell pellet. In the following, a final concentration of 0.5 μg/mL in 50 μL of RNase A stock solution was added to samples and were then incubated at 4 °C for at least 4 h. 106 events from stored samples kept at 4 °C were examined using flow cytometry^25^.

### Scratch assay for cell migration analysis

MDA-MB 231 cells 2 × 10^5^ cell/well were seeded in 6-well plates and allowed to adhere and grow to 70–80% confluence. A sterile 1000-μL micropipette tip was used to scratch the cell monolayer in each well. After scratching, cellular debris was removed by washing the wells once with 1 ml of the culture medium, and smoothed edges of the scratched cell were achieved. Next, 5 mL of culture medium containing the peptides and Doxorubicin at IC_50_ and IC_25_ concentrations were added to each well, and the plate was incubated for 24 h.

With the help of a micro-meter blade, the distances were measured for imaging, and the outside bottom of the plate was marked with tip marks that approximated the scratch. Ultimately, the reference mark is left outside the capture image region while the plate is inspected under a phase-contrast microscope. ImageJ software was used to measure the cell migration distance after images were taken at regular intervals of 24 h up to 72 h. Each scratch closure's distance was calculated as determined by the cell front and measured by comparing the photos taken at time 0 with those taken at different time points.

### Real-time PCR

At first, 1 × 10^5^ MDA-MB 231 cells/well were cultured in a 12-well microplate. Next, nrCap18 peptide was added to the wells at IC_50_ concentration and treated for 48 h. After 48 h, total RNA was extracted according to the RNA isolation kit protocol. Additionally, the cDNAs were synthesized by a cDNA synthesis kit. Then, Real-time PCR strips were mixed with cDNA, CDK4 and CDK6 primers, and the Real-time PCR Master Mix. The LightCycler® 96 Instrument (Roche, Basel, Switzerland) was then used to run them through 40 cycles of 30 S at 95 °C, 30 S at 60 °C (annealing temperature), and 30 S at 72 °C. The results were interpreted using the 2^−∆∆Ct^ technique. Relative CDK4/6 (Cyclin-Dependent Kinases 4/6) gene expression levels were normalized by GAPDH (Glyceraldehyde 3-phosphate dehydrogenase). The primer sequences are shown in Table 1.^26^

**Table 1:** Sequence of primers.

Gene	Sequence	Length	Tm (°C$$)$$
CDK4	Forward: 5′-CTGGTGTTTGAGCATGTAGACC-3′	22	55.41
	Reverse: 5′-GATCCTTGSTCGTTTCGGCTG-3′	21	56.39
CDK6	Forward: 5′-CCAGSTGCTCTAACCTCAGT-3′	21	54.71
	Reverse: 5′-AACTTCCACGAAAAAGAGGCTT-3′	22	54.8
GAPDH	Forward: 5′-ACAACTTTGGTATCGTGGAAGG-3′	22	54.71
	Reverse 5′-GCCATCACGCCACAGTTTC-3′	19	55.28

### Statistical analysis

All data from this study were presented as mean with standard deviation (mean ± SD). GraphPad Prism 9.0 was used to perform all statistical analysis. One-way and two-way ANOVA were used to find the significant differences between the means. In cases where the probability value obtained was less than 0.05 (*P* < 0.05), the differences were considered significant.

## Results

### Cytotoxicity of nrCap18 on MDA-MB 231 and MCF-10A cell lines

**Figure 1:**
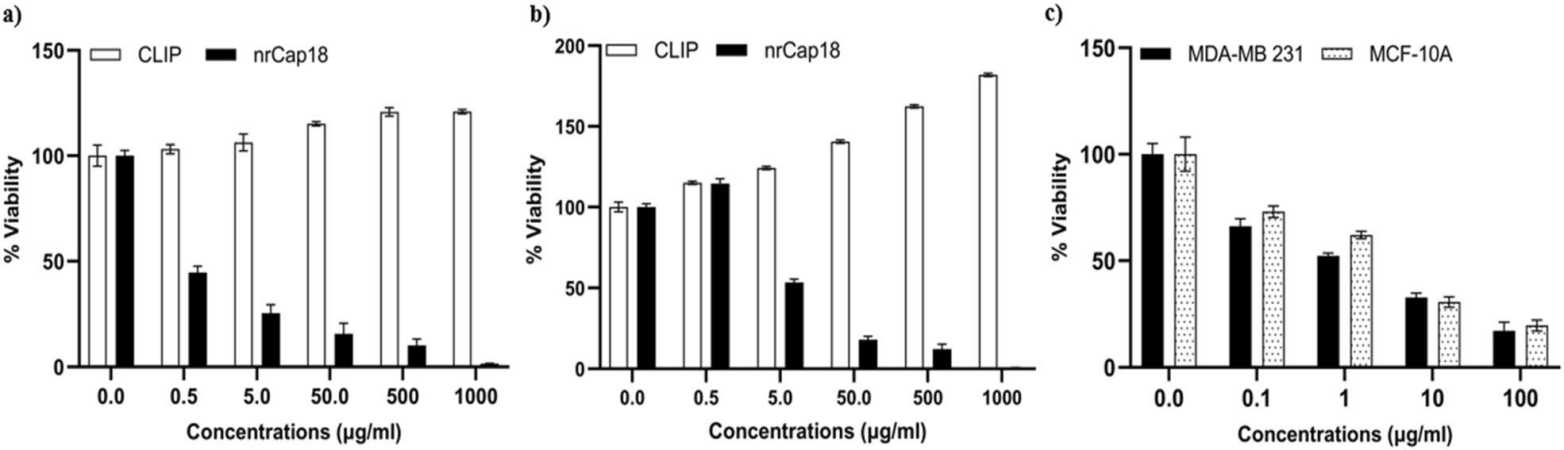
The viability/concentration charts. The results indicate a dose-dependent increase in cytotoxicity of nrCap18 against cells. (**a**) MDA-MB 231 cell line, (**b**) MCF-10A cell line, and (**c**) DOX cytotoxicity comparison in both cell lines. DOX: doxorubicin.

The toxicity of nrCap18 and Doxorubicin toward the cancer cell line (MDA-MB 231) was evaluated by MTT assay. Based on the viability/concentration curve, the IC_50_ for the peptide was obtained by GraphPad Prism 9.0 software. Results show that compared to CLIP (a non-cytotoxic peptide), the nrCap18 peptide exhibited highly significant cytotoxicity on MDA-MB 231 (Fig. 1a). In addition, the results showed that the cytotoxicity of nrCap18 on MCF-10A, as a normal cell line, is significantly less than its potential cytotoxicity on the cancer cell line (Fig. 1b). Also, the results showed doxorubicin cytotoxicity percent against the MDA-MB 231 and MCF-10A cells has no significant difference (Fig. 1c).

**Figure 2:**
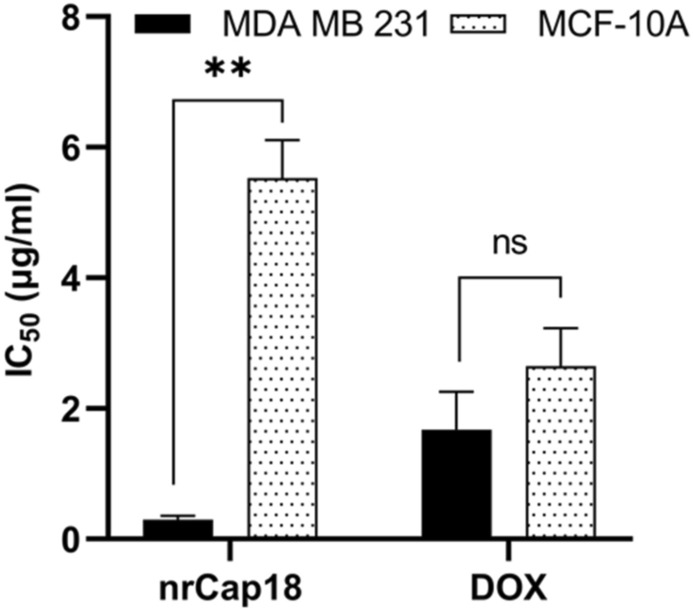
Comparing the IC_50_ values of nrCap18 and DOX in MDA-MB 231 and MCF-10A cell lines. DOX: doxorubicin. ns *P* > 0.05 and **P < 0.01. DOX: doxorubicin.

The IC_50_ for nrCap18 on MDA-MB 231 was approximately 0.29 μg/ml, while this value for MCF-10A was 5.53 μg/ml. The IC_50_ of the mentioned peptide in MCF-10A was significantly more than MDA-MB 231 (*P* < 0.01). Also, the value of IC_50_ for Doxorubicin against MDA-MB 231 and MCF-10A was 1.6 and 2.65 μg/ml, respectively (Fig. 2).

### Selectivity of nrCap18 for *cancer* cells

The selectivity of nrCap18 for cancer cells (MDA-MB 231) was investigated by comparing the cytotoxicity of this peptide by MTT assay after washing method in both the breast normal and cancerous cell lines.

In the MDA-MB 231 cell line, the peptide showed a selective and potent effect on cells in the microplate and was not removed by washing. The peptide's cytotoxicity was compared in a new viability/concentration curve.

**Figure 3:**
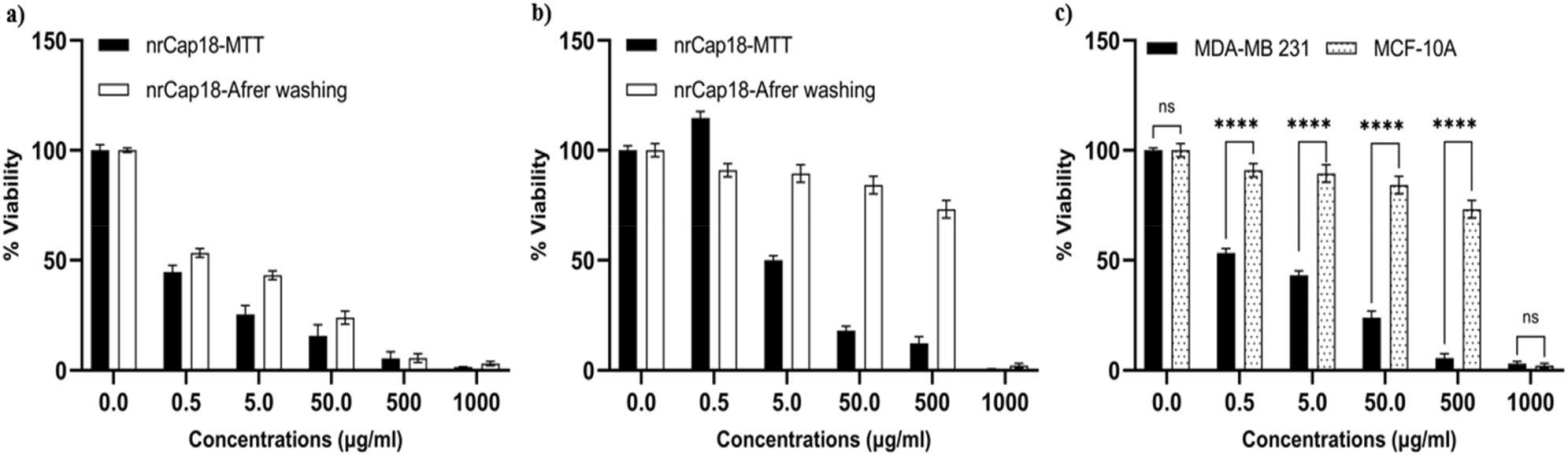
The MTT after washing viability/concentration charts. The comparison of viability of (**a**) MDA-MB 231 and (**b**) MCF-10A cell lines in standard MTT and MTT after washing methods. (**c**) Shows the viability percentage of MDA-MB-231 and MCF-10A after washing the nrCAp18 peptide. ns *P* > 0.05 and ****P < 0.0001.

In the MCF-10A cell line, this cationic peptide would be removed easily by washing cells after 1 h of treatment. The viability/concentration curve confirms this explanation (Fig. 3).

### Using flow cytometry to measure apoptosis

**Figure 4:**
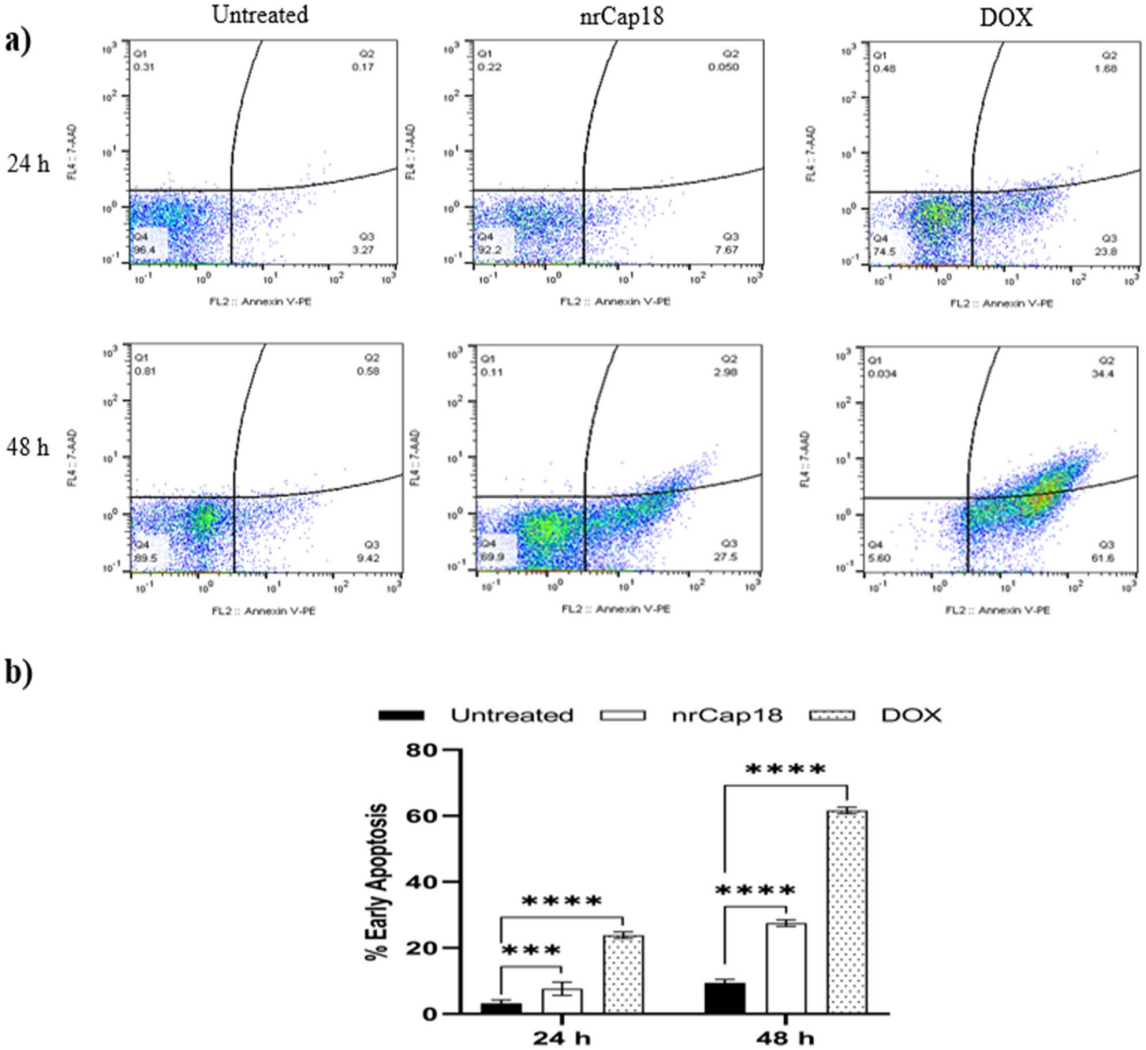
(**a**) Displays dot plot charts illustrating the results of apoptosis/necrosis analyzed via flow cytometry at two distinct time points. (**b**) The percentage of early apoptotic MDA-MB 231 cells in untreated and cells treated with nrCap18 and DOX at two different times. DOX: doxorubicin. ***P < 0.001, ****P < 0.0001.

Using flow cytometry data and dot plot charts, a graph was drawn that demonstrates how nrCap18 affected the apoptotic status of MDA-MB 231. As can be seen, nrCap18 increased the early apoptosis rate significantly compared to the untreated group (*P* < 0.05) in time (Fig. 4).

### AO/EB double staining

**Figure 5:**
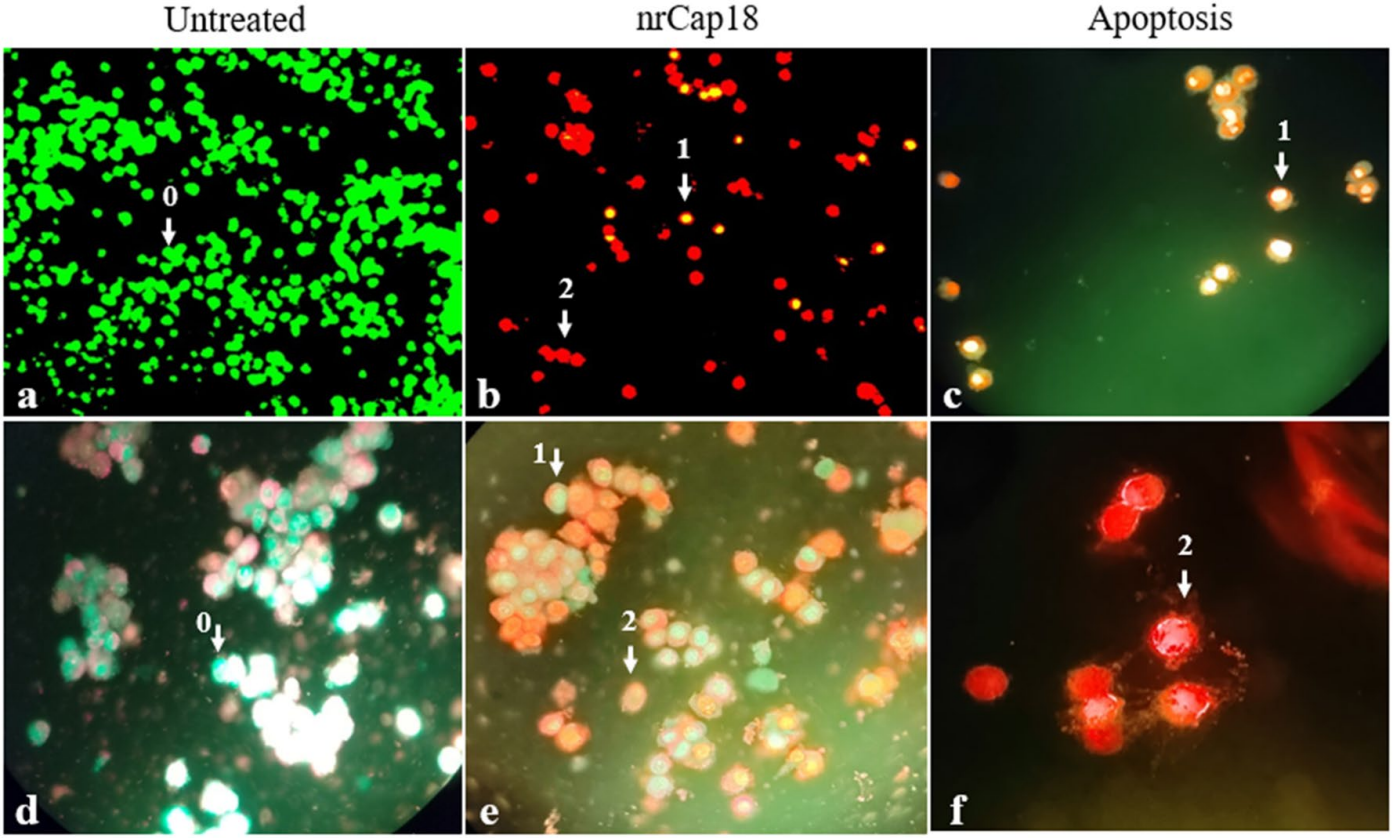
AO/EB double staining assay. This figure shows fluorescent images of MDA-MB 231 cells following treatments with IC_50_ of nrCap18 after 72 h. Images (**a** and **b**) were taken by inverted microscope while direct microscopy took images (**c** to **f**). The untreated cells (**a** and **d**) are negative control (without peptide treatment), while **b** and **e** are nrCap18 treated cells. Parts (**c** and **f)** demonstrated different stages of apoptosis. **0:** Live cell, **1:** Early apoptosis, **2:** Late apoptosis. (**a** and **b**) images were taken with a 20 × lens, and (**c** to **e**) images with a 40 × lens. Image (**f**) was taken with a 100 × lens. The images were selected with the best views.

MDA-MB 231 cells were labeled by AO/EB after applying 72 h treatment. Dual staining was examined under a fluorescent microscope—early-stage apoptotic cells, marked by green-yellow acridine orange nuclear staining. Late-stage apoptotic cells were recognized by concentrated and red–orange nuclear ethidium bromide staining. Meanwhile, normal living cells appeared to show green fluorescence (Fig. 5).

### Analysis of cell cycle arrest

**Figure 6:**
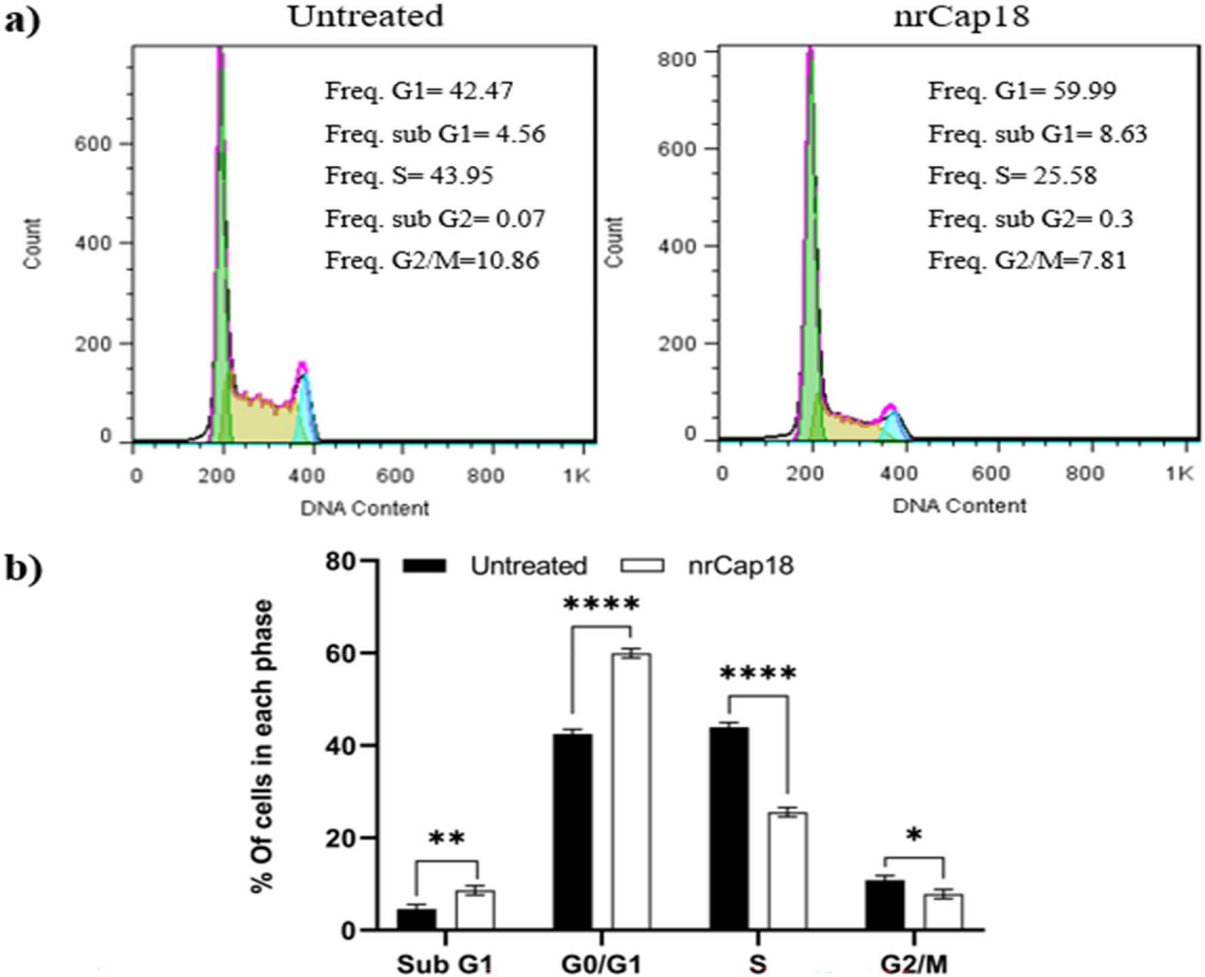
The Cell Cycle arrest results. (**a**) Cell cycle status for untreated MDA-MB-231 cells and cells treated by nrCap18. (**b**) The percentage of cell population in different cell cycle phases. *P < 0.05, **P < 0.01, ****P < 0.0001.

MDA-MD 231 cells were treated at IC_50_ of nrCap18. After 48 h of treatment, a significant increase in the G0/G1 phase (approximately 30%) and a decrease showed in their presence in the S phase compared to negative control (*P* < 0.0001), which signifies a rise in apoptosis, a halt in the cell cycle in the G0/G1 phase, and a reduction in DNA-replicating cells (Fig. 6).

### Wound-healing assay for cell migration

**Figure 7:**
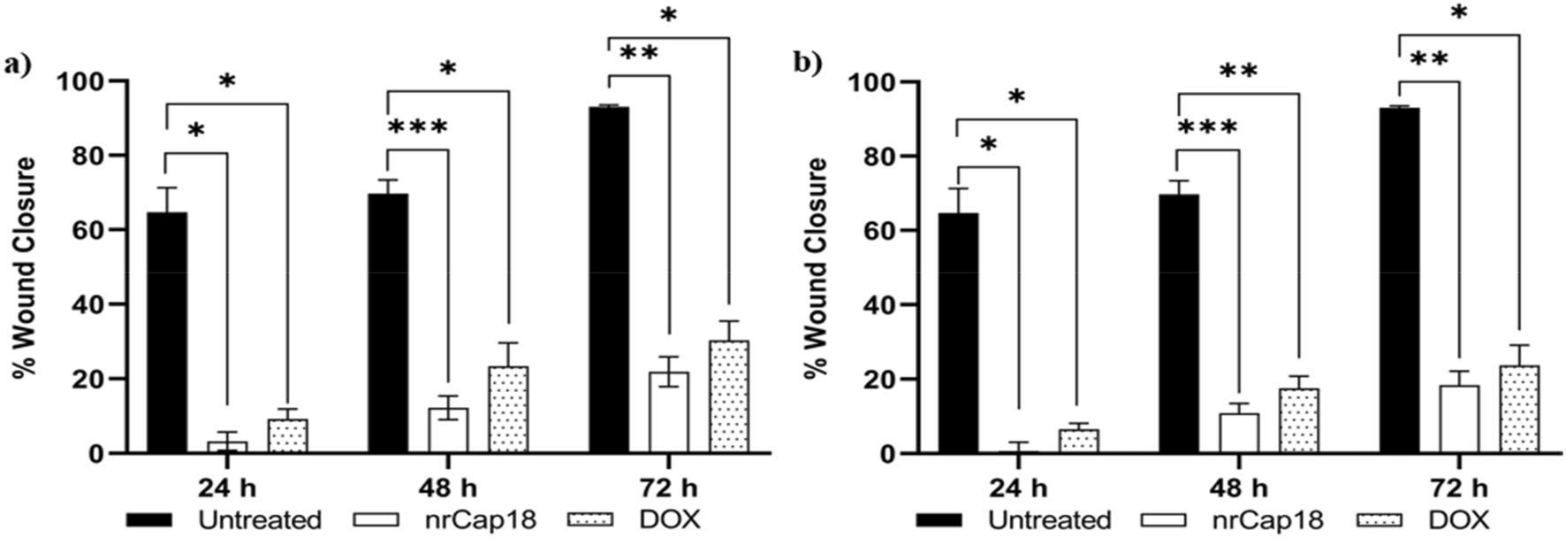
The effect of nrCap18 and DOX at (**a**) IC_25_ and (**b**) IC_50_ concentrations on MDA-MB 231 cell migration and invasion potential. DOX: doxorubicin. *P < 0.05, **P < 0.01, ***P < 0.001.

**Figure 8:**
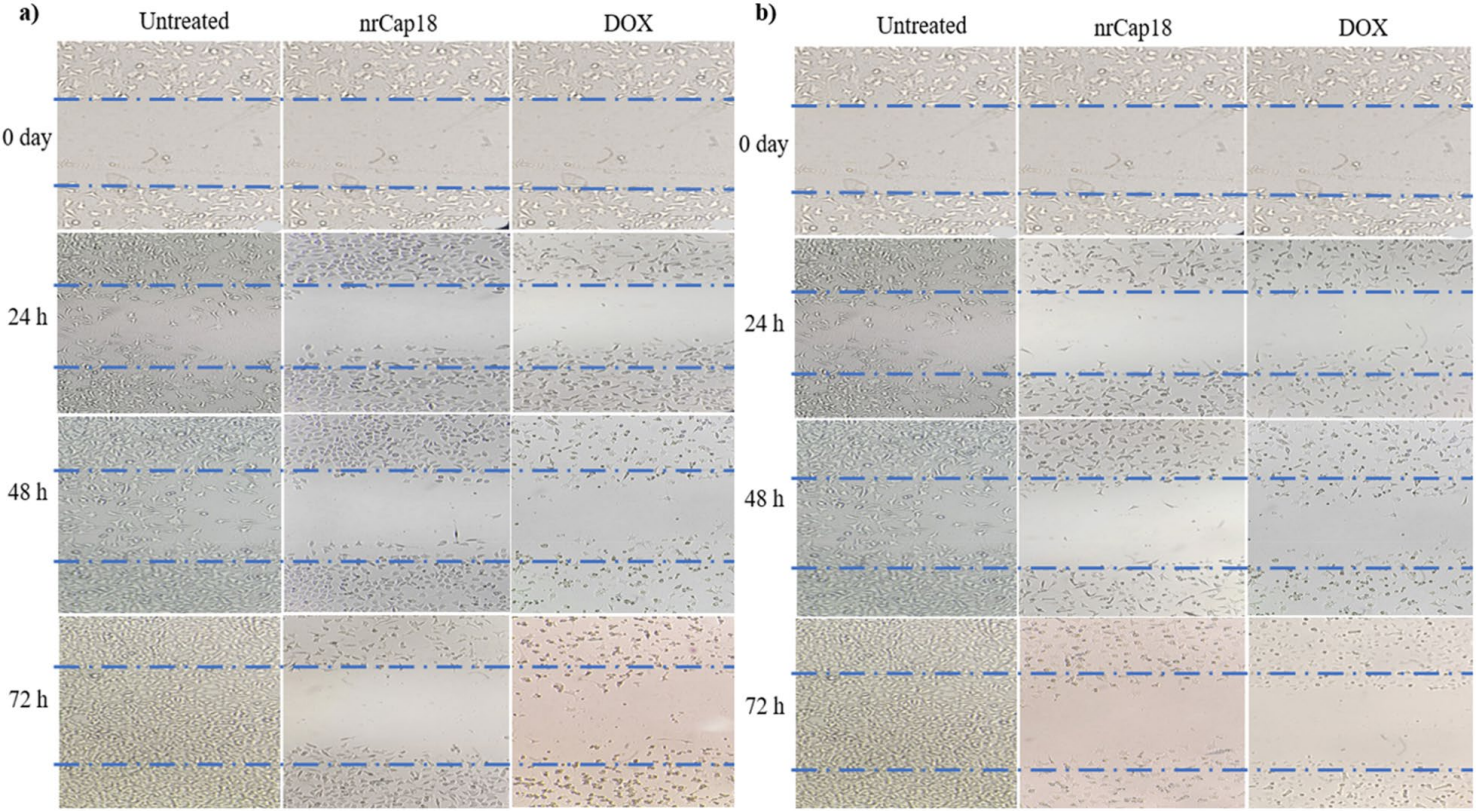
Representing images were captured at three distinct time points and depict the restraining effect of individual compounds (**a**) IC_25_ and (**b**) IC_50_ concentration on the migration of cancer cells. DOX: doxorubicin. Images were taken with a 20 × lens.

Results showed that the treatment at IC_25_ and IC_50_ significantly reduced the cell migration capacity of MDA-MB231 (Fig. 7). As demonstrated in (Fig. 8), the scratch remained left unfilled following peptide treatment; in contrast, after 72 h, untreated control cells completely migrated and filled the gap. Images were captured at regular intervals of 24 h, and ImageJ was used to quantify the area between each cell, as shown in Fig. 8.

### Effect of nrCap18 on CDK4 and CDK6 expression

**Figure 9:**
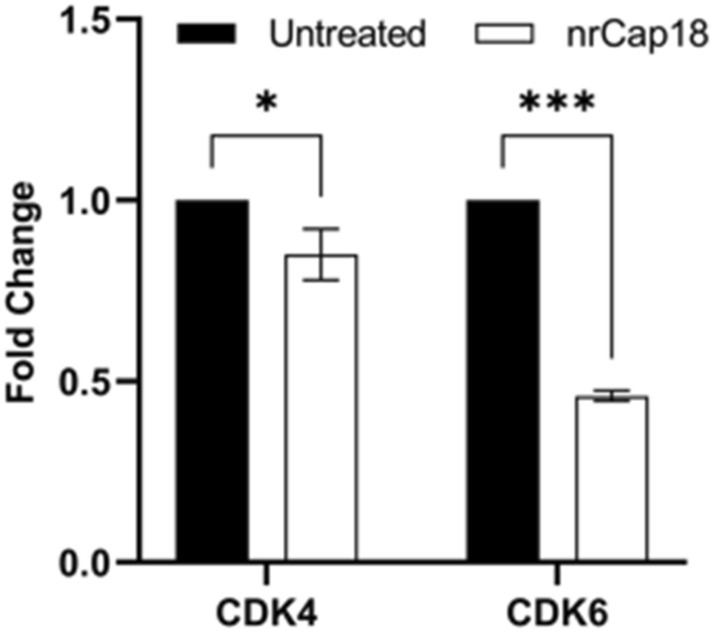
Real-time PCR results. Relative CDK4/6 gene expression after the mentioned treatment. As the graph shows the amounts of CDK4/6 genes due to treatment with nrCap18 have decreased significantly compared to the untreated group. *P < 0.05, *** *P* < 0.001.

The quantitative analysis of CDK4 and CDK6 relative expression compared to GAPDH expression demonstrated a noteworthy reduction in the expression of the target genes (CDK4 and CDK6) when the mentioned peptide was added to MDA-MB 231 cells. Specifically, the expression levels decreased significantly, reaching 0.9 (*P* < 0.05) and 0.46 (*P* < 0.001) compared to the control group (Fig. 9).

## Discussion

The promising rise and achievement in cancer therapy over the past decade has developed the clinical management of many malignancies that were previously endowed with poor outcomes^1^. Triple-negative breast cancer (TNBC), recognized as a particularly aggressive and highly metastatic tumor with limited response to chemotherapy, carries the poorest prognosis. Surgery and chemotherapy are currently the two most prominent treatment options available for TNBC^2^.

Recent research suggests that focusing on the interaction between the innate immune system and cancer may hold great promise for treating tumors^27,28^. Within this concept, therapeutic peptides have emerged as a distinct category of pharmaceutical agents and have garnered significant attention in pharmaceutical research. Recent advancements in peptide chemistry technologies, specifically in protein purification, synthesis, structure elucidation, and sequencing, have been pivotal in driving progress in this field^29^. Consequently, the development of peptide drugs has gained momentum, with approximately 40 peptide drugs receiving global approval. It is worth mentioning that synthetic peptides have also been developed^30^.

Since the early discovery of therapeutic peptides, they have shown great potential in various fields, such as hormones, growth factors, neurotransmitters, ion channel ligands, and anti-infective agents. These peptides bind to receptors on the surface of cells, triggering specific and highly effective intracellular reactions. Interestingly, they share a similar mode of action with other biologics, such as therapeutic proteins and antibodies. Nevertheless, therapeutic peptides exhibit lower immunogenicity and are more cost-effective than biologics^31–33^. Additionally, several studies have identified peptides capable of inducing cell death in breast cancer cells^31,34–36^.

Based on the cumulative evidence, it can be inferred that peptides like Cathelicidin-derivative and its fragments possess advantageous properties and demonstrate strong biological interactions. Hence, they hold promise as potential compounds for cancer therapies. These peptides could be a novel platform for developing anti-tumor drugs, particularly for tumors that resist existing treatment methods^37,38^.

Madanchi et al., in their research^39^, presented a group of modified compounds derived from cathelicidin, including nrCap18, as peptides possessing antimicrobial properties. In light of the specific characteristics of this peptide, our current study aims to investigate its potential as an anti-cancer agent. Hence, assuming the possible anti-cancer effects of nrCap18 and taking into account the limitations of chemotherapy and the relatively high prevalence of triple-negative breast cancer (TNBC), we employed nrCap18 on the MDA-MB 231 cell line, known as a triple-negative cell line, to elucidate some of its anti-cancer aspects.

Based on the present study, it has been determined that nrCap18 exhibits an anti-cancer effect. When testing on the MDA-MB 231 cell line, the viability decreased dose-dependent upon treatment with nrCap18. The IC_50_ values for nrCap18 and doxorubicin were approximately 0.29 μg/ml and 1.6 μg/ml, respectively. The cytotoxicity of nrCap18 on normal human breast cells, the MCF-10A cell line, was also utilized. The IC_50_ value for nrCap18 on MCF-10A cells was calculated to be 5.23 μg/ml. Madanchi and colleagues reported an IC_50_ value of approximately 335 μg/ml for Hu02 cells^39^. The lower IC_50_ value of nrCap18 on the MCF-10A cells compared to the Hu02 cells can be attributed to its heightened sensitivity. In general, these findings exhibit coherence and stability.

The dysregulation of apoptosis in cancer cells allows them to survive longer. This extended survival allows for the accumulation of mutations, which can enhance tumor progression, stimulate the growth of new blood vessels (angiogenesis), disrupt normal control of cell growth, and hinder the process of cell differentiation. Generally, cancer cells employ various strategies to evade apoptosis, which renders them resistant to many pro-apoptotic agents, including certain anti-cancer drugs^40^. When comparing the effects of nrCap18 to doxorubicin, it was found that nrCap18 resulted in a lower rate of early apoptosis during 24 and 48 h of incubation. However, a notable observation was made at the 48-h mark, where the rate of early apoptosis induced by nrCap18 significantly increased and approached the rate observed with doxorubicin at 24 h. The increasing effects suggest that the effectiveness of nrCap18 may improve over time, with its maximum effects potentially occurring after 24–48 h, as shown in the AO/EB test with 72 h incubation. Some specific peptides, such as BMAP-28^41^, have been shown to induce cell death by causing a disruption in membrane polarity, ultimately leading to necrosis.

In contrast, our observations indicate that nrCap18, at its IC_50_ dosage, primarily induces early apoptosis, whereas in high doses, especially 1 mg/ml induces necrosis rather than early apoptosis. These findings suggest that nrCap18 may have a distinct mechanism of action compared to BMAP-28 and Doxorubicin. Therefore, further studies are needed to elucidate the molecular mechanisms behind the differential apoptotic effects of nrCap18 and to explore its potential as an anti-cancer therapy.

The uncontrolled growth of cells is a prominent feature of cancer, and the maintenance of telomere length mainly facilitates this. Consequently, there is considerable interest in targeting the mechanisms responsible for telomere maintenance as a therapeutic approach^42^. In a recent study about a newly presented peptide named W12K^43^, it was found that there is a difference between cells treated with the peptide and control-treated cells. The analysis revealed an escalation of the G1 and S phases in the W12K-treated cells, leading to a corresponding decline in the G2M phase. Furthermore, using the nrCap18 peptide in cell treatment has been shown to alter the cell cycle pattern in the MDA-MB 231 cancer cell line. This alteration is characterized by an increase in the population of cells in the G1 and SubG1 phases, indicating the induction of apoptosis and a decrease in cells in the G2/M phase, suggesting an inhibitory effect on cell proliferation. The arrest during the G1 phase of cell division, irrespective of ER, HER2, and p53 status, triggers apoptosis by inhibiting telomeres^44^. This inhibition also effectively prevented cancer from spreading to other body parts (metastasis). Furthermore, multiple studies have recognized that the arrest in both the G1 and Sub G1 phases is associated with increased gene transcription that promotes cell death (p73, p21, Bax, Bad)^45^.

The successful therapeutic targeting of metastatic cancer necessitates addressing the dynamic plasticity of cancer cells as they navigate the process of metastasis, as well as the strategies employed by dormant and growing metastases to manipulate their surrounding habitats and avoid detection by the immune system. Among the last new strategies, checkpoint immunotherapy has a limited impact on metastatic cancers, especially breast cancer, and the average 5-year survival rate remains an obstacle for patients diagnosed with the recurrent form of this disease^46^. Treating metastasis, therefore, needs alternative therapy. A study examining the impact of LL-37, compared to LL-25, on the migration of MCF-7 breast cancer cells reported that 2 μM LL-37 resulted in triple the number of migrating MCF7 cells compared to the control group. Consistent with in vivo data, adding LL-25 (1 μM) neutralized the migratory effect of LL-37, confirming its inhibitory potential. However, LL-25 alone did not significantly affect cell migration^41^. Fortunately, our current study demonstrated that nrCap18 and doxorubicin strongly hindered cancer cell migration at IC_25_ and IC_50_ concentrations. Even after 72 h of incubation, the cell wound remained unfilled, as observed. In contrast, the control group of cells migrated entirely and filled the wound gap. However, due to the limited knowledge regarding the pathophysiological traits of metastases compared to primary tumors, it becomes imperative to explore novel strategies for managing and halting their progression^47^.

The cyclin D1-CDK4/6 complex plays a significant role in cellular mechanisms by directly phosphorylating and stabilizing the transcription factor known as FOXM1. This phosphorylation process leads to the activation of FOXM1, which in turn promotes the progression of the cell cycle. Moreover, it also protects cancer cells, preventing them from entering a state of senescence. Furthermore, this complex is involved in the phosphorylation and inactivation of SMAD3, which mediate the anti-proliferative response induced by TGF-β^48^. Important mediators that help cells enter the S phase, CDK4 and CDK6 are essential for the start, development, and survival of numerous cancer kinds. The current standard of therapy for patients with advanced hormone receptor-positive breast cancer has efficiently shifted to comprise pharmaceutical compounds that target CDK4/6^49^. As expected, CDK4/6 inhibitors arrest sensitive tumor cells in the G1 phase of the cell cycle. However, the effects of CDK4/6 inhibition reach far beyond this initial action^50^. Based on our investigation into peptide treatment, a noteworthy reduction in the expression levels of CDK4 and CDK6 genes was observed. These findings provide support for the cessation of the G1 Cell cycle data. Conversely, previous research suggests that halting at the G1 Cell Cycle Phase does not rely on the presence of the 3 markers (ER, HER2, P53)^51^ and thus could be efficacious for treating negative triple breast cancers. Hence, this peptide may exhibit effectiveness in treating negative triple breast cancers.

## Conclusion

Our findings indicate that the nrCap18 peptide has a specific cytotoxic effect on cancer cells. Our investigation also revealed that this peptide can hinder the migration of cancer cells by disturbing the cell cycle. Further in-depth and meticulous research is essential to fully comprehend the mechanisms and potential adverse effects of this peptide. Nevertheless, this peptide harbors promising potential as a novel anticancer peptide. But surely more pre-clinical and clinical studies should be done to reach this claim.

## Data Availability

All data and materials are available in the article. Other data will be provided upon request.
